# Is S-upar level correlated to the length of hospitalization

**DOI:** 10.1186/1757-7241-23-S1-A32

**Published:** 2015-07-16

**Authors:** Esben K Baymler, Ina K Jensen, Mathias B Danielsen, Astrid J Damgaard, Kjeld A Damgaard

**Affiliations:** 1 Emergency Medicine Department, Hospital of Vendsyssel, Denmark

## Background

The purpose of this study is to investigate if S-uPAR correlates with expected length of patients' hospitalization. This could be useful in emergency medicine, due to the acute departments treating patients hospitalized for 48 hours or less. S-uPAR (Urokinase Plasminogen Activator, CD87) is a potential biomarker thought to be related to inflammatory immune cell activation. It is expressed on various immune cells including neutrophils, monocytes, macrophages, and lymphocytes. Upon inflammation, it is cleaved from the cell surface and released into serum. S-uPAR has shown prognostic and clinical value in the triage of patients as described in an editorial comment in the Journal of Internal Medicine 2012 [1].

## Methods

It is a follow-up study including 60 unselected patients (n = 60) above 60 years of age and is the first sample of a larger study including 500 patients. Blood samples in this study have been collected from patients at admission. The samples were frozen afterwards and the patients have been examined retrospectively by physicians and stratified into groups: hospitalized < 24 hours, 24-72 hours, and > 72 hours.

## Results

The mean S-uPAR value for the < 24h group (n = 14) was 2.75 (1.5 - 24.3). This compared to the 24-72h group (n = 13) with a mean value of 4.3 (3.4-16.9) and the > 72h group (n = 29) with a mean value of 4.1 (0.8-18.7).

## Conclusion

Patients hospitalized < 24h have lower S-uPAR values compared to patients hospitalized >24h. Preliminary findings of the study show no correlation between the patients S-uPAR values and the length of hospitalization. When all 500 patients have been included, further analysis will be conducted, including analysis of patients presenting very high and very low S-uPAR values to exclude those with known confounders.
